# Cohesion Fatigue Explains Why Pharmacological Inhibition of the APC/C Induces a Spindle Checkpoint-Dependent Mitotic Arrest

**DOI:** 10.1371/journal.pone.0049041

**Published:** 2012-11-07

**Authors:** Pablo Lara-Gonzalez, Stephen S. Taylor

**Affiliations:** Faculty of Life Sciences, University of Manchester, Manchester, United Kingdom; Mayo Clinic, United States of America

## Abstract

The Spindle Assembly Checkpoint (SAC) delays the onset of anaphase in response to unattached kinetochores by inhibiting the activity of the Anaphase-Promoting Complex/Cyclosome (APC/C), an E3 ubiquitin ligase. Once all the chromosomes have bioriented, SAC signalling is somehow silenced, which allows progression through mitosis. Recent studies suggest that the APC/C itself participates in SAC silencing by targeting an unknown factor for proteolytic degradation. Key evidence in favour of this model comes from the use of proTAME, a small molecule inhibitor of the APC/C. In cells, proTAME causes a mitotic arrest that is SAC-dependent. Even though this observation comes at odds with the current view that the APC/C acts downstream of the SAC, it was nonetheless argued that these results revealed a role for APC/C activity in SAC silencing. However, we show here that the mitotic arrest induced by proTAME is due to the induction of cohesion fatigue, a phenotype that is caused by the loss of sister chromatid cohesion following a prolonged metaphase. Under these conditions, the SAC is re-activated and APC/C inhibition is maintained independently of proTAME. Therefore, these results provide a simpler explanation for why the proTAME-induced mitotic arrest is also dependent on the SAC. While these observations question the notion that the APC/C is required for SAC silencing, we nevertheless show that APC/C activity does partially contribute to its own release from inhibitory complexes, and importantly, this does not depend on proteasome-mediated degradation.

## Introduction

The spindle assembly checkpoint (SAC) ensures accurate chromosome segregation during mitosis by delaying the onset of anaphase until all the chromosomes are attached to the mitotic spindle via their kinetochores [1]. When kinetochores are not correctly attached to the spindle, they activate the SAC pathway, producing a diffusible inhibitor that targets the Anaphase Promoting Complex/Cyclosome (APC/C), an E3 ubiquitin ligase that promotes the proteasome-mediated degradation of several anaphase inhibitors, including cyclin B and securin [2], [3]. It is generally accepted that the inhibitor corresponds to the mitotic checkpoint complex (MCC), composed of BubR1, Bub3, Mad2 and the APC/C co-activator Cdc20 [4]. Once all the chromosomes are attached, the SAC is silenced and this leads to APC/C activation; cyclin B1 and securin are then degraded, promoting anaphase onset and mitotic exit [1].

Multiple mechanisms have been proposed to mediate SAC silencing. These include dynein-dependent stripping of kinetochore proteins [5] and the activation of phosphatases that counteract the activity of mitotic kinases [6]. Recently, p31^comet^ has been shown to be required for MCC disassembly downstream of kinetochores [7], [8]. Specifically, p31^comet^ promotes the release of Mad2 from the MCC, contributing to a first step for MCC disassembly [9]. This mechanism acts mostly on free MCC, but not on the fraction of the MCC that is bound to the APC/C (APC/C^MCC^). How then Mad2-free MCC and APC/C^MCC^ disassemble is currently unclear.

One mechanism might involve APC/C ubiquitylation activity. Several lines of evidence indicate that the APC/C promotes SAC silencing by promoting the ubiquitylation and degradation of an unknown factor [10], [11], [12], [13], [14], [15]. Accordingly, when cells are released from a mitotic arrest into the proteasome inhibitor MG132, the MCC cannot disassemble, despite the presence of apparently normal kinetochore-microtubule attachments [16], [17], thus indicating that the SAC cannot be silenced when proteolysis is blocked. Other observations seem to contradict this however; for example, when SAC signalling was silenced by treating cells with a combination of taxol and an Aurora B inhibitor, the MCC disassembled, even when proteasome activity was blocked [16], [17], [18], [19]. These complexities suggest that perhaps some sort of feedback loop exists between the SAC and ubiquitylation-dependent degradation [17].

Key evidence in favour of this model comes from the use of a small molecule APC/C inhibitor, tosyl-L-arginine methyl ester (TAME) [13]. TAME inhibits APC/C activity by blocking the binding of co-activators via their IR tails [13]. In addition, a cell permeable pro-drug, proTAME, was shown to cause a prolonged metaphase arrest in cells, consistent with APC/C inhibition. Strikingly however, this arrest was dependent on a functional SAC pathway [13]. This result is at odds with the notion that the APC/C is downstream of the SAC; a mitotic block caused by direct APC/C inhibition should not be affected by inhibition of upstream SAC components. Indeed, depletion of Cdc20 or inhibition of the proteasome with MG132 has been shown to block mitosis in a SAC-independent manner [13], [20]. Nevertheless, it was argued that if the APC/C is required for SAC silencing, then partial APC/C inhibition with proTAME might cause a mitotic arrest by blocking SAC silencing, suggesting the existence of a positive feedback loop between the SAC and the APC/C. While provocative, this model requires further testing.

We therefore set out to use proTAME as a tool to further explore the possibility that APC/C activity is required for SAC silencing. Consistent with the original report [13], we found proTAME does induce a mitotic arrest that is indeed SAC-dependent. However, we show that this can simply be explained by the induction of cohesion fatigue, a recently described phenomenon whereby sister chromatids separate during a prolonged metaphase arrest [21], [22]. This is caused by the microtubule pulling forces during metaphase that can eventually overcome the cohesin-based forces that hold sister chromatids together. Because cohesion fatigue generates unpaired sisters that cannot stably attach microtubules, the SAC is re-activated [22]. Thus the SAC-dependent arrest induced by proTAME is an indirect effect of cohesion fatigue.

## Results

### proTAME Induces Prolonged Mitotic Arrest Following Cohesion Fatigue

**Figure 1:**
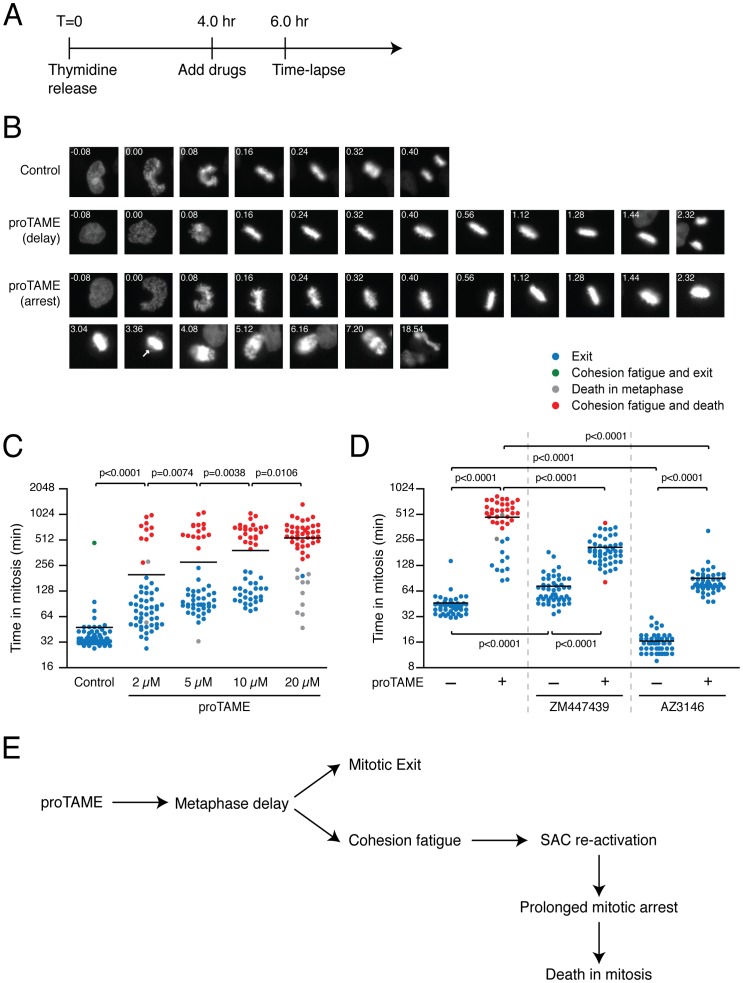
proTAME induces cohesion fatigue and prolonged mitotic arrest. (**A**) Timeline showing the experimental strategy. HeLa cells were synchronised by a double thymidine block and then released into normal media for 4 hours before the addition of drugs. (**B**) Time-lapse sequences indicating the different phenotypes of cells treated with 10 µM proTAME. Numbers indicate the time from nuclear envelope breakdown (NEBD) in hours:minutes and the arrow points the appearance of cohesion fatigue. (**C, D**) Scatter plot showing the amount of time cells take from NEBD to mitotic exit after treatment with different concentrations of proTAME (C) or upon treatment with 15 µM proTAME, in combination with the Aurora B inhibitor ZM447439 or the Mps1 inhibitor AZ3146 (D). The horizontal line represents the mean and the colours represent the cell fate (see legend). At least 40 cells were analysed in each condition. P values were calculated using two-tailed Mann-Whitney test. (**E**) Model explaining how proTAME can cause a SAC-dependent mitotic arrest.

In order to study the role of APC/C activity in SAC silencing, we tested the ability of proTAME to arrest cells in mitosis. HeLa cells stably transfected with GFP-histone H2B were synchronised in S-phase, released into media containing proTAME at different concentrations and analysed by time-lapse microscopy (Fig. 1A). Whereas control cells completed mitosis normally, proTAME induced two very different phenotypes. Some cells exited mitosis apparently normally after a brief metaphase delay (Fig. 1B). In others, the metaphase delay appeared to be followed by a gradual loss of sister chromatid cohesion (Fig. 1B), a phenomenon previously described as cohesion fatigue [21], [22]. Initially, only a few chromosomes detached from the metaphase plate but eventually many chromosomes moved to the spindle poles. These cells arrested in mitosis for many hours until they finally died (Fig. 1B). At lower doses of proTAME, a mixed phenotype was apparent whereas at higher doses most cells underwent cohesion fatigue, mitotic arrest and cell death (Fig. 1C and S1). To test whether the proTAME-induced arrest was dependent on an active SAC, synchronised cells were released into media containing proTAME plus one of two drugs known to override the SAC, namely the Aurora B inhibitor ZM447439 [23], or the Mps1 inhibitor AZ3146 [24]. In both cases, the brief mitotic delay still manifested, but the prolonged mitotic arrest followed by cell death was avoided (Fig. 1D). Similar results were obtained when the SAC was inhibited through BubR1 or Mad2 depletion (Fig. S2).

At face value, these results suggest that the metaphase delay induced by proTAME is due to a direct inhibition of the APC/C (Fig. 1E). Eventually, cells enter anaphase and exit mitosis normally. However, if cells are delayed in metaphase for long enough, then cohesion fatigue is triggered, and this in turn re-establishes the SAC. In these circumstances, penetrant inhibition of the APC/C is mediated by the SAC, independently of proTAME (Fig. 1E). In other words, the mitotic arrest followed by cell death appears to be an indirect consequence of cohesion fatigue. This provides a simpler explanation for why the proTAME-induced mitotic arrest is also dependent on the SAC.

### proTAME-induced Cohesion Fatigue Causes SAC Re-activation

**Figure 2:**
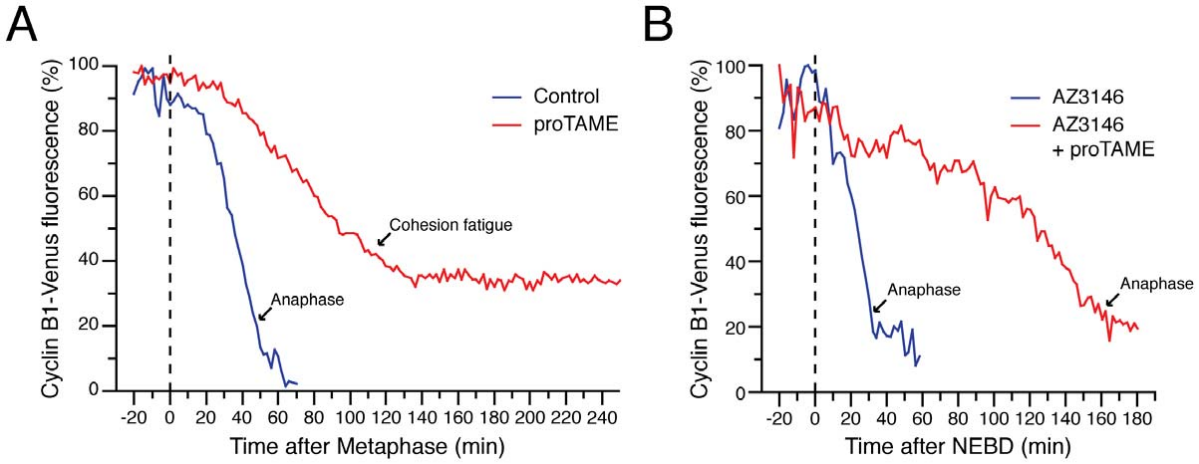
proTAME induces SAC re-activation following loss of sister chromatid cohesion. (**A, B**) Graphs measuring cyclin B fluorescence intensity upon proTAME treatment (5 µM), either in the absence (A) or presence (B) of AZ3146. In each case, T = 0 was normalised to the onset of metaphase or prometaphase respectively. Arrows indicate the time at which anaphase or cohesion fatigue occurs. Data from one representative cell is shown for each treatment.

Our data suggests that proTAME-induced cohesion fatigue causes SAC re-activation. To test this, we monitored cyclin B degradation kinetics. HeLa cells were co-transfected with plasmids encoding cyclin B-Venus and histone H2B-DsRed and then analysed by time-lapse microscopy. Control cells initiated degradation of cyclin B at metaphase, as described previously [25]. In cells treated with proTAME, cyclin B degradation started at metaphase, although with slower kinetics, consistent with proTAME inhibiting APC/C activity. However, when these cells underwent cohesion fatigue, cyclin B degradation stopped (Fig. 2A), thus suggesting that the SAC is being re-activated under these conditions. When we inhibited the SAC with AZ3146, cyclin B degradation started at prometaphase, again consistent with previous observations [25]. When cells were treated with AZ3146 and proTAME, cyclin B degradation started at prometaphase, but again with slower kinetics - presumably due to APC/C inhibition. However, because these cells did not spend enough time in mitosis for cohesion fatigue to manifest, degradation continued until the cells finally entered anaphase and exited mitosis (Fig. 2B). Similar results were obtained when securin degradation was analysed (data not shown). Thus, these results show that the SAC is indeed reactivated when proTAME-treated cells undergo cohesion fatigue, providing a simple explanation as to why the prolonged arrest induced by proTAME is SAC-dependent.

### Inhibition of Cohesion Fatigue Resists proTAME-induced Mitotic Arrest

**Figure 3:**
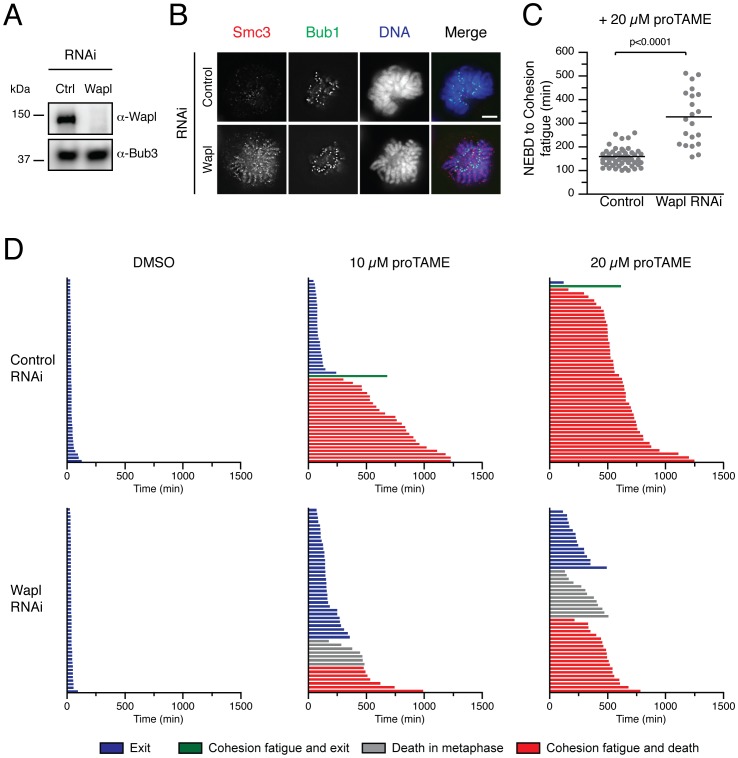
Increasing the level of cohesin on the chromosomes inhibits the mitotic arrest caused by proTAME. (**A**) Immunoblot of HeLa cells treated showing Wapl depletion upon RNAi treatment. Bub3 was used as a loading control. (**B**) Immunofluorescence images of mitotic HeLa cells showing an increased amount of the cohesin subunit Smc3 on the chromosomes upon Wapl depletion. (**C**) Scatter plot of HeLa cells depleted of Wapl and treated with proTAME, showing an increase in the amount of time cells take to undergo cohesion fatigue. P values were calculated using two-tailed Mann-Whitney test. (**D**) Cell fate profiles of HeLa cells depleted of Wapl and treated with different concentrations of proTAME, as described in Fig. 1A. Each line represents an individual cell and different colours represent the different cell fates (see legend).

Taking these results together, a simpler mechanism emerges: proTAME induces partial APC/C inhibition that delays the metaphase to anaphase transition. However, if cohesion fatigue occurs during this delay, then the SAC is re-activated. Because single sisters cannot stably attach microtubules, the SAC cannot now be satisfied, and thus a prolonged mitotic arrest is imposed. If this is the case, then inhibition of cohesion fatigue should modify cell behaviour after proTAME treatment: rather than arresting and dying, cells should more likely delay and exit mitosis. To test this, we depleted Wapl by RNAi (Fig. 3A). Wapl is a protein involved in removing cohesin from chromosome arms during prophase [26], [27], and inhibition of Wapl resists cohesion fatigue [21], [22]. Consistent with previous observations [26], [27], Wapl RNAi increased the amount of cohesin on chromosomes during prometaphase, as indicated by increased immunostaining for the cohesin subunit Smc3 (Fig. 3B). Importantly, when we exposed cells to proTAME and considered only those that underwent cohesion fatigue, Wapl RNAi significantly extended the time before it manifested, indicating that inhibition of Wapl does indeed strengthen cohesive forces (Fig. 3C). When considering the populations as a whole, in order to better visualise the data, we plotted “*cell fate profiles*” [28], in which each horizontal line represents one cell with the length of the line representing the duration of mitosis and the colour of the line indicating cell fate (Fig. 3D). Wapl RNAi-treated cells progressed through mitosis normally in the absence of drugs (Fig. 3D, left panels). However, in the presence of proTAME, depletion of Wapl shifted the cell fate profile such that now more cells exited mitosis while less underwent cohesion fatigue and death (Fig. 3D, middle and right panels). Specifically, in 10 µM proTAME, 52% of cells underwent anaphase and exited mitosis, while 46% underwent fatigue and death. In Wapl-RNAi cells treated with 10 µM proTAME, 70% of cells underwent exit, while 16% exhibited fatigue and death. At 20 µM proTAME, in this particular experiment, only one cell (2%) underwent exit, but Wapl-RNAi increased this to 33%. Thus, by resisting cohesion fatigue, cells are more likely to undergo a brief metaphase delay followed by a relatively normal anaphase and mitotic exit, and less likely to undergo a prolonged mitotic arrest and cell death.

### MCC Disassembly does not Require Proteasome Activity

The original proTAME data showing a SAC-dependent mitotic arrest was used to support the notion that APC/C activity is required to silence the SAC [13]. However, our data indicate that the SAC-dependent mitotic arrest caused by proTAME is due to cohesion fatigue. Therefore, we decided to further investigate the role of the APC/C-proteasome pathway in SAC silencing.

For instance, it was recently shown that cells released from a mitotic arrest into the proteasome inhibitor MG132 still contain high amounts of assembled MCC [16], [17], suggesting that proteasome activity is required for MCC disassembly, and therefore by extension for SAC silencing. However, we were surprised by these results because we previously showed that when the SAC is satisfied by treating cells with a combination of taxol and an Aurora B inhibitor, the MCC dissociates from the APC/C, even when the proteasome is inhibited [18]. Because proteasome inhibition can also cause cohesion fatigue [21], [22], and in light of the proTAME data described above, we asked whether cohesion fatigue might explain this discrepancy.

**Figure 4:**
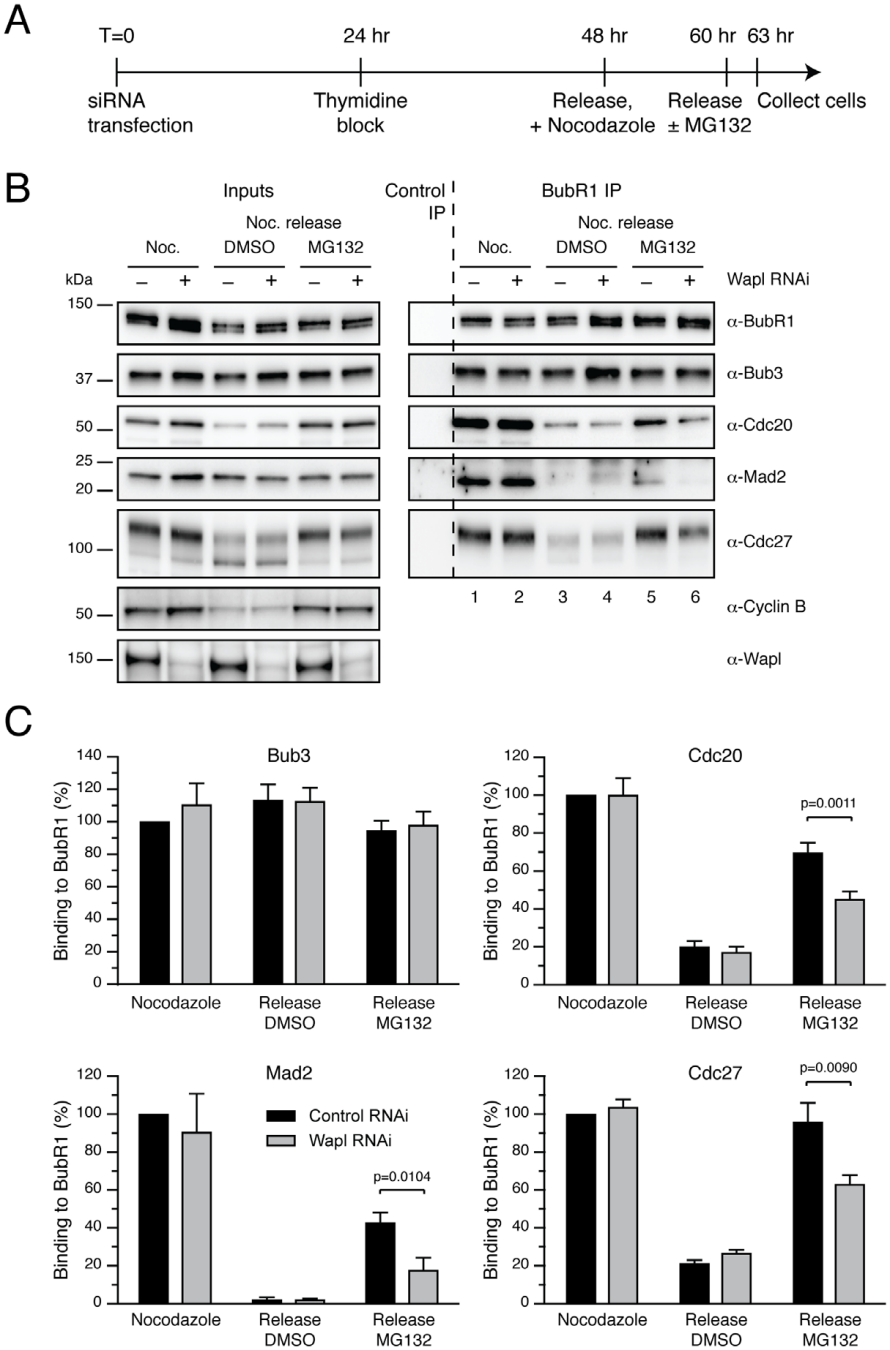
Inhibition of cohesion fatigue promotes MCC disassembly, even when the proteasome is inhibited. (**A**) Timeline showing the experimental strategy. (**B**) Immunoblots of BubR1 immunocomplexes isolated from HeLa cells. Cells were treated with Wapl RNAi, synchronised with a single thymidine block and then released into media containing nocodazole for 12 hours before being selectively detached and released into normal media or media containing MG132 for 3 hours. Cyclin B was used as a mitotic marker. (**C**) Bar graph quantifying the experiment in (B). Values represent the mean ± s.e.m. of three independent experiments, each analysed twice. P values were calculated using paired, two-tailed *t*-test.

To test this, nocodazole-arrested HeLa cells were released into normal media or media containing MG132 (Fig. 4A). To assess MCC levels, BubR1 was immunoprecipitated and the levels of co-precipitating Bub3, Cdc20, Mad2 and APC/C analysed by quantitative immunoblotting. When control mitotic cells were released into drug-free media, the levels of Cdc20, Mad2 and APC/C co-purifying with BubR1 were severely reduced, (Fig. 4B, compare lanes 1 and 3. Note that Bub3 remains bound to BubR1 during mitotic exit), indicating MCC disassembly and APC/C dissociation following mitotic exit. However, when cells were released into media containing MG132, MCC levels remained high (Fig. 4B, compare lanes 1 and 5), consistent with previous observations [16], [17]. To test whether cohesion fatigue might account for this, we depleted Wapl. Under these conditions, MCC disassembly was partially rescued in the presence of MG132 (Fig. 4B, compare lanes 5 and 6; and 4C for quantification). This suggests that the persistence of MCC levels in the presence of MG132 can be explained, at least in part, by SAC re-activation following cohesion fatigue. (Note that the partial effect is readily explained by the fact that Wapl RNAi does not completely block cohesion fatigue (Fig. 3D) and see refs [21], [22]).

**Figure 5:**
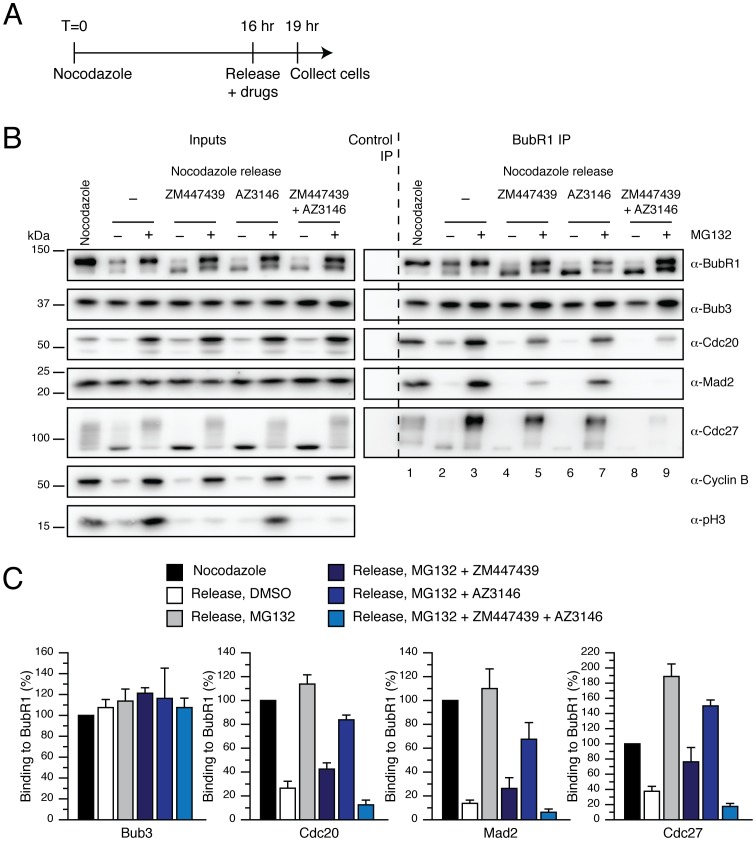
Proteasome activity is not required for MCC disassembly. (**A**) Timeline showing the experimental strategy. (**B**) Immunoblot of BubR1 immune complexes isolated from HeLa cells arrested in mitosis with nocodazole and released into media containing different combinations of MG132, ZM447439 and AZ3146. Cyclin B and phosphorylated histone H3 Ser10 (pH3) were used as markers for mitosis and Aurora B activity respectively. (**C**) Bar graph quantifying the experiment in (B). Values represent the mean ± s.e.m. of three independent experiments.

To confirm that proteasome activity is not required for MCC disassembly, we harvested nocodazole-arrested cells, released them into MG132 and added ZM447439 or AZ3146 to prevent SAC signalling. MCC formation and APC/C binding were then analysed by performing BubR1 immunoprecipitations. When either ZM447439 or AZ3146 were added, partial MCC disassembly occurred in the presence of MG132 (Fig. 5A, compare lanes 3, 5 and 7; and B for quantification). Because these treatments are predicted to only partially block SAC activity [29], we released cells from a nocodazole block into media containing MG132 and both, ZM447439 and AZ3146 [29]. Under these conditions, only residual amounts of MCC were detectable (Fig. 5A, compare lanes 3 and 9; and B for quantification). Similar results were obtained when we used alternative Aurora B and Mps1 inhibitors, such as Hesperadin and Reversine (data not shown). Thus, when the SAC is silenced, pre-assembled MCC can disassemble and dissociate from the APC/C despite the fact that the proteasome is blocked. Because binding of the MCC to the APC/C is the key step in maintaining a SAC-dependent mitotic block, these data indicate that proteasome activity is not required for SAC silencing. This is entirely consistent with our previous observation [18] and strongly suggests that the more recent data showing that MG132 inhibits MCC disassembly [16], [17] arise due to SAC re-activation following cohesion fatigue.

### APC/C Activity is Required for MCC Disassembly

**Figure 6:**
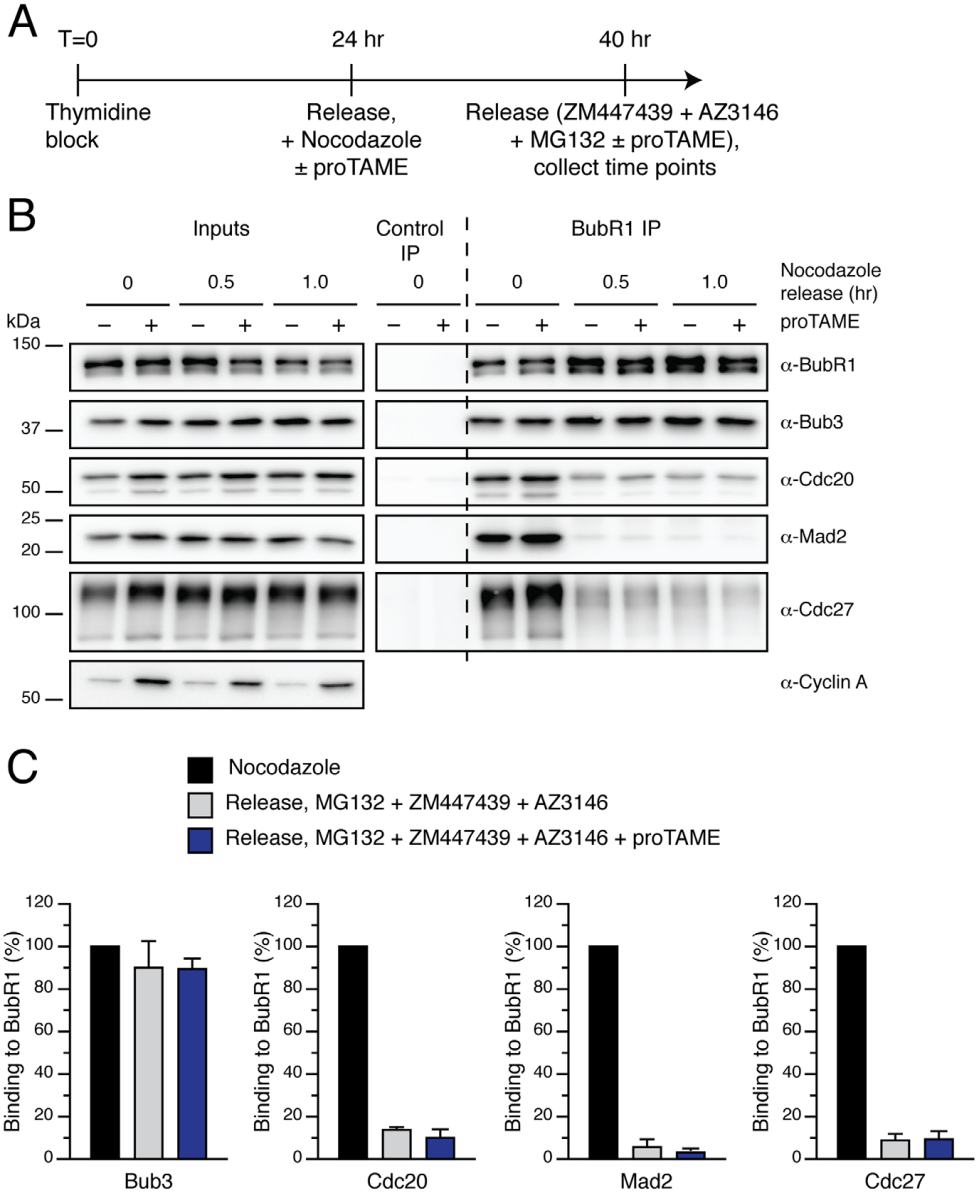
proTAME treatment does not prevent MCC turnover. (**A**) Timeline showing the experimental strategy. (**B, C**) Immunoblots of BubR1 immune complexes isolated from mitotic HeLa cells arrested with nocodazole and released into media containing MG132, ZM447439 and AZ3146, in the presence or absence of 20 µM proTAME. Cyclin A was used as a marker for lack of APC/C activity. (**C**) Bar graph quantifying the experiment in (B). Values represent the mean ± s.e.m. of three independent experiments.

While our results show that proteasome activity is not required for MCC disassembly, it remains possible that APC/C ubiquitylation activity is required for SAC silencing, independent of proteolytic degradation, as previously suggested [11], [12]. To test this, HeLa cells were released from a nocodazole block into media containing MG132, ZM447439 and AZ3146, either in the presence or absence of proTAME (Fig. 6A). Cells were then collected and BubR1 was immunoprecipitated. Under these conditions, proTAME significantly inhibited APC/C activity, as evidenced by increased stability of cyclin A (Fig. 6B). However, addition of proTAME did not affect MCC disassembly (Fig. 6B and C for quantification).

The simplest explanation for these results is that APC/C activity is not essential for MCC disassembly. However, this could reflect the fact that proTAME is not a very potent APC/C inhibitor (Fig. 2A and B) [13]. In addition, it was shown recently that, upon TAME treatment, substrates can still be ubiquitylated by the APC/C but not in a very efficient manner and thus are not targeted for degradation [30]. Therefore, if only partial ubiquitylation activity was required for MCC disassembly, then this might not be evident by using proTAME.

**Figure 7:**
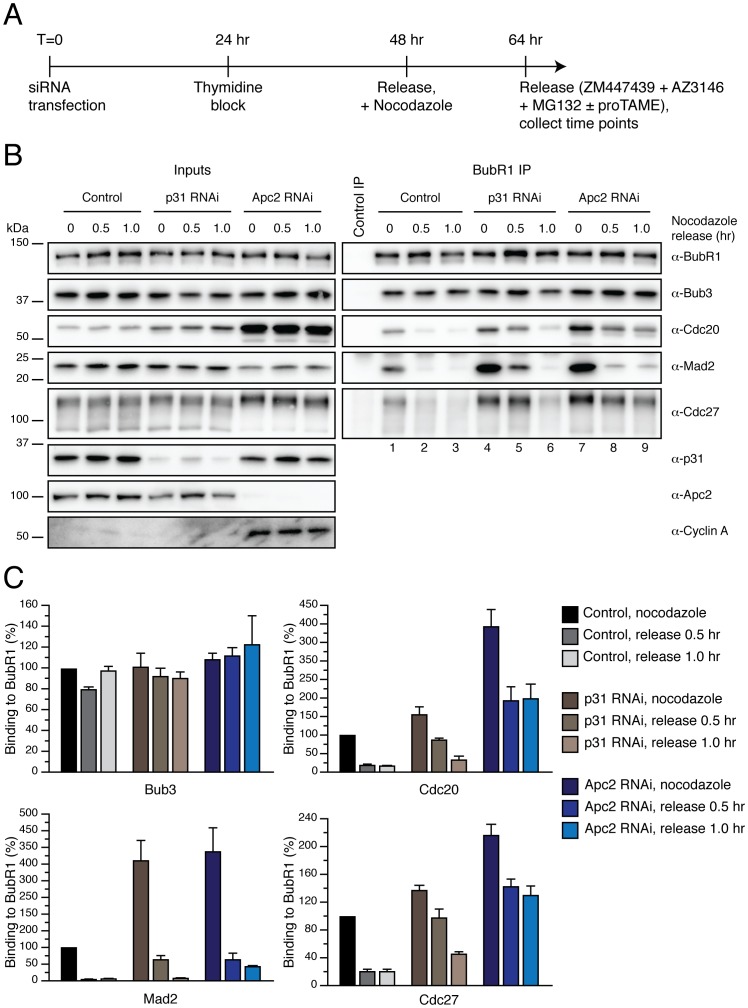
APC/C activity is required for MCC disassembly independently of proteolysis. (**A**) Timeline showing the experimental strategy. (**B**) Immunoblots of BubR1 immunocomplexes isolated from mitotic HeLa cells depleted of p31^comet^ or Apc2 by RNAi, arrested in mitosis with nocodazole and released into media containing MG132, ZM447439 and AZ3146. Cyclin A was used as a marker for lack of APC/C activity. (**C**) Bar graph quantifying the experiment in (B). Values represent the mean ± s.e.m. of four independent experiments.

In order to induce a more penetrant block on APC/C ubiquitylation activity, we repeated our nocodazole release experiments following RNAi to deplete the catalytic APC/C subunit Apc2 [31]. As a positive control, we depleted p31^comet^, which has previously been shown to contribute to disassembly of APC/C-free MCC [7], [8], [9], [10], [11] (Fig. 7A). Apc2 RNAi led to significant stabilisation of both Cdc20 and cyclin A, suggesting efficient APC/C inhibition (Fig. 7B). When we performed a nocodazole release, Apc2 RNAi lead to significantly higher levels of MCC in mitosis; note more Mad2 and Cdc20 bound to BubR1 at T_0_ (lanes 1 vs. 7). Additionally, 1 hour after release, MCC levels remained higher (Fig. 7B, lanes 3 vs. 9; and C for quantification). Note also that upon Apc2 depletion, MCC proteins still bind the remaining, Apc2-free APC/C. This suggests that APC/C activity might indeed be required for MCC disassembly.

Interestingly, the effect of inhibiting p31^comet^ was slightly different: the MCC did still disassemble but at a slower rate when compared to control cells (Fig. 7B, lanes 2 vs. 5; and C for quantification). Thus, this raises the possibility that p31^comet^ and APC/C activity act on different pathways to promote MCC disassembly. Indeed, we recently showed that p31^comet^ acts on MCC that is not bound to the APC/C [9], raising the possibility that the APC/C-dependent pathway promotes disassembly of APC/C-bound MCC.

### APC/C Activity is Required to Dissociate the MCC from the APC/C

As alluded to above, p31^comet^ is required for MCC disassembly but only acts on free MCC, not on APC/C-bound MCC. Thus, we hypothesised that APC/C ubiquitylation activity is required for releasing the MCC from the APC/C, thus explaining the different phenotypes obtained when we depleted p31^comet^ or Apc2 (Fig. 7). To test this, we set out to separate the free-MCC from the APC/C-bound MCC fractions in order to analyse whether depletion of Apc2 had an effect on disassembly of either or both.

**Figure 8:**
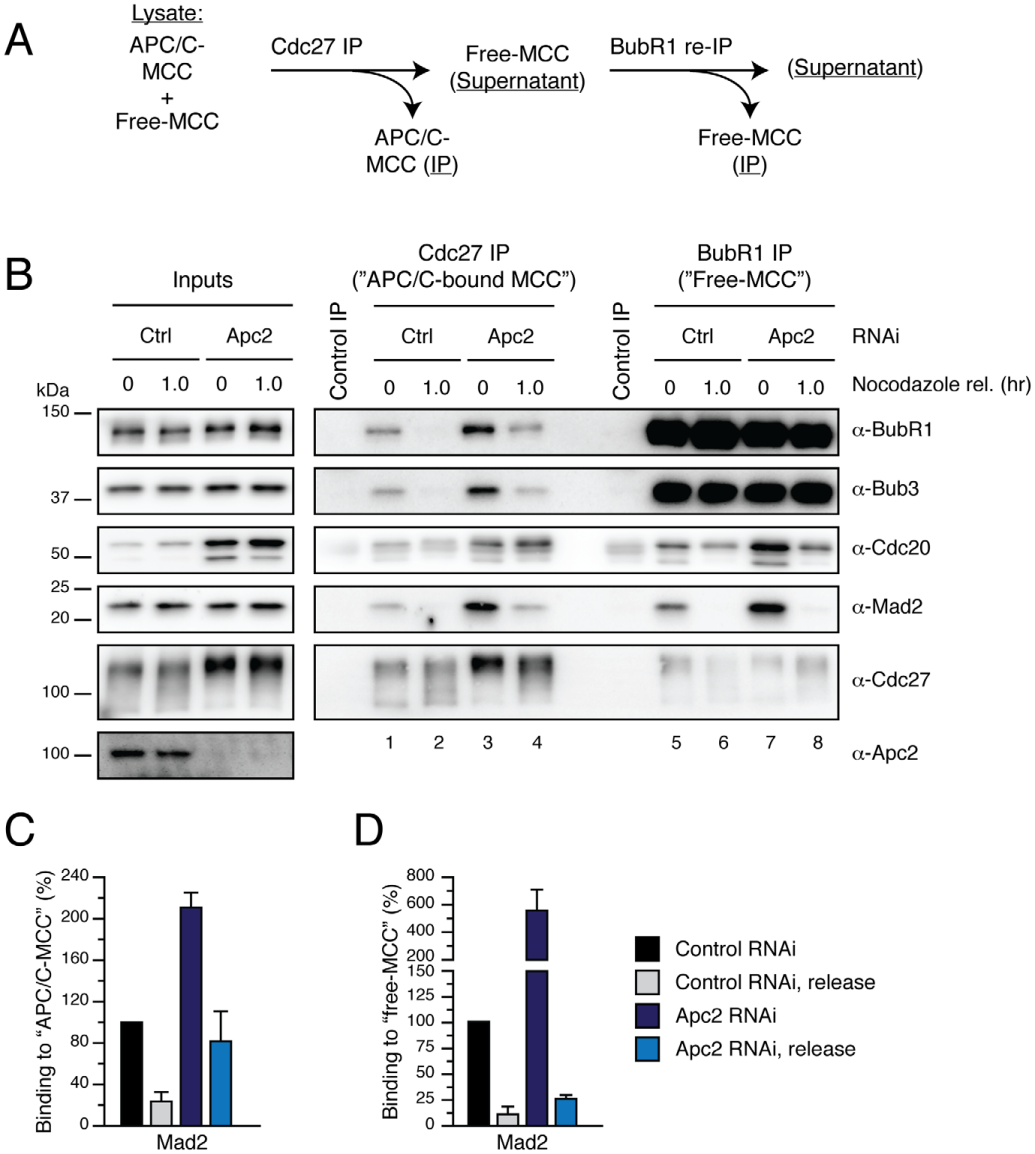
APC/C activity is required for releasing itself from the MCC. (**A**) Schematic representation of the strategy for the selective isolation of APC/C-MCC and free MCC. (**B**) Immunoblot of immunocomplexes isolated from mitotic HeLa cells released into media containing MG132, ZM447439 and AZ3146 after Apc2 RNAi. (**C, D**) Bar graph quantifying the amount of Mad2 bound to APC/C-MCC (C) or free-MCC (D) under the different conditions described in (A). Values represent the mean ± s.e.m. of three different experiments.

For this, we first performed a Cdc27 immunoprecipitation in order to analyse APC/C^MCC^, and then immunoprecipitated BubR1 from the remaining supernatant of these samples in order to analyse APC/C-free MCC (Fig. 8A). Under these conditions, increased amounts of BubR1, Bub3, Cdc20 and Mad2 were bound to the APC/C when Apc2 was depleted (Fig. 8B, compare lanes 1 and 3; and C for quantification). Upon nocodazole release, significant levels of these proteins remained bound to the APC/C in Apc2 RNAi-treated cells (Fig. 8B, compare lanes 2 and 4; and C for quantification). When we analysed the effect of Apc2 RNAi on free-MCC, we noted that Apc2 RNAi also lead to an increase in the amount of Cdc20 and Mad2 bound to BubR1 (Fig. 8B, compare lanes 5 and 7; and D for quantification). However, when cells were released from the nocodazole arrest, similar levels of Mad2 were obtained bound to BubR1 in Apc2 RNAi when compared to control-treated cells (Fig. 8B, compare lanes 6 and 8; and D for quantification). Taken together, these results suggest that APC/C ubiquitylation activity contributes to the release of the MCC proteins from the APC/C and, importantly, this does not require proteasome-dependent degradation.

## Discussion

The APC/C has been proposed to promote SAC silencing by ubiquitylation of one or more unidentified factor [10], [11], [12], [13], [14]. A key supporting observation comes from the use of an APC/C inhibitor, proTAME, that surprisingly caused a SAC-dependent mitotic arrest, evoking the existence of a negative feedback loop between the SAC and the APC/C [13]. However, here we provide a much simpler explanation for this surprising result, namely that the SAC-dependent mitotic arrest induced by proTAME is an indirect effect caused by cohesion fatigue.

Many treatments that delay cells in metaphase have been shown to cause cohesion fatigue, regardless of their mechanism of action. These include proteasome inhibition, expression of non-degradable cyclin B, expression of a spindly F258A mutant, depletion of Cdc20 or Ska3, and inhibition of Cenp-E [20], [21], [22], [32], [33]. Thus, it should not come as a surprise that proTAME can also induce cohesion fatigue. Our data shows that proTAME does inhibit APC/C activity, yielding a metaphase delay, and that, if during this delay cohesion fatigue occurs, the SAC is re-activated thus imposing a prolonged mitotic arrest.

In addition to APC/C activity, the proteasome has also been proposed to be required for SAC silencing and/or MCC disassembly. Accordingly, when mitotic cells are treated with the proteasome inhibitor MG132, more MCC is evident [10], [17], [34]. However, this might simply reflect the fact that proteasome inhibition stabilises Cdc20; as *de novo* synthesis creates more Cdc20, it is mopped up by Mad2, thereby creating more MCC (Fig. S3). Despite this, additional lines of evidence have been used to argue that proteasome inhibition blocks MCC disassembly [16], [17]. Here we provide evidence indicating that this can also be explained by the induction of cohesion fatigue (Fig. 4). Moreover, when SAC re-activation following cohesion fatigue is blocked, the MCC can disassemble, regardless of whether the proteasome is inhibited or not (Fig. 5). Thus, proteasome activity is not a primary determinant in SAC silencing.

Despite these issues, proTAME (and MG132) is a useful tool for studying APC/C-activity; our observations confirm that proTAME does inhibit the APC/C in cells. However, when designing and interpreting experiments to study SAC silencing, the spectre of cohesion fatigue has to be taken into account. Note that the use of other chemical biology tools does make it possible to create experimental conditions to study SAC silencing while at the same time avoiding the cohesion fatigue problem (Fig. 5). For example, the use of an Aurora B inhibitor in conjunction with Taxol will stabilize kinetochore-microtubule interactions and thereby satisfy the SAC while at the same time diminishing the microtubule pulling forces necessary for cohesion fatigue.

While we show that proteolysis is not required for SAC silencing, our data supports the notion that APC/C activity is required for promoting the dissociation of the MCC from the APC/C in a proteasome-independent manner, consistent with previous suggestions [11], [12]. Moreover, our data show that at least two mechanisms are at play; while p31^comet^ acts by promoting the disassembly of free MCC [9], ubiquitylation contributes to the dissociation of the MCC from the APC/C. However, it must be noted that the APC/C can still dissociate from the MCC, even when APC/C activity is greatly inhibited (Fig. 7 and 8), suggesting that either spontaneous dissociation occurs or that there are an additional mechanisms.

Whether APC/C ubiquitylation activity alone is sufficient for MCC disassembly [11] or this is aided by another factor is not clear. Recently, it was shown that the small APC/C subunit Apc15/Mnd2 was required for MCC disassembly and SAC silencing, despite being dispensable for APC/C activity [12], [15]. In addition, the ubiquitin binding protein CUEDC2 was shown to be required for the disassembly of the Cdc20-Mad2 complex [35]. Perhaps these mechanisms act on the same pathway as APC/C ubiquitylation activity to promote the release of the MCC from the APC/C. Further work will be required to test this possibility.

However, this model induces a paradox: if MCC binding blocks APC/C activity, how can the APC/C promote the ubiquitylation of factors that will promote its own activation? On the other hand, under conditions in which the SAC is active, substrates such as Cyclin A and Nek2A can still be ubiquitylated [36], indicating that, under those conditions, the APC/C is still active towards some proteins. An attractive possibility if that the factor ubiquitylated by the APC/C is the inhibitor itself. Such a mechanism has been proposed for another APC/C inhibitor, XErp1/Emi2 [37].

Accordingly, Cdc20 is ubiquitylated by the APC/C during mitosis [38], [39] with initial studies showing that Cdc20 ubiquitylation promoted its dissociation from Mad2 [11]. However, non-ubiquitylable versions of Cdc20 can still dissociate from the MCC and the APC/C [12], [38]. Also, even though APC/C activity is required for SAC silencing in *Xenopus* egg extracts, this does not seem to depend on Cdc20 ubiquitylation [37]. Furthermore, a Cdc20 version that lacks all its lysine residues overrides the SAC in the presence of spindle toxins, suggesting that Cdc20 ubiquitylation is required for maintaining the SAC [15], [38].

Another candidate is BubR1. BubR1 is an APC/C substrate *in vitro* and in cells [40], [41]. In addition, recent work shows that BubR1 acetylation protects it from being recognised by the APC/C as a substrate during a mitotic arrest. However, when the SAC is silenced, BubR1 becomes ubiquitylated and this promotes SAC silencing [41]. Further identification and characterisation of the APC/C targets that promote SAC silencing will certainly provide insights into how the SAC activating and silencing signals are balanced in mitosis, which can have implications for the development of chemotherapeutic drugs, since mitotic exit has been proposed to be a very effective target for the treatment of cancer [20], [42].

## Methods

### Cell Culture and Drug Treatments

Parental HeLa and HeLa GFP-H2B cells were as described [18], [43]. For synchronising cells in S-phase, cells were treated with 2 mM Thymidine (Sigma) for 16 hours, then released into normal media for 8 hours and treated with thymidine for further 16 hours. To synchronise cells in mitosis, cells were treated with 1 µM nocodazole for 16 hours.

proTAME was synthesised by Peakdale and used in an assay-dependent concentration (2–20 µM). Initial experiments comparing this compound and proTAME kindly provided by Randy King showed that both were able to induce a very similar mitotic delay in cells (Fig. S1D). MG132 (Sigma) was used at 20 µM and ZM447439 (Tocris) and AZ3146 (a gift from AstraZeneca) were at 2 µM.

### RNAi

For RNAi treatments, HeLa cells were transfected with 50 nM of the siRNA sequences (Dharmacon) using Interferin (PolyPlus). The siRNA sequences used were: BubR1 VJ4 (5′-AACGGGCAUUUGAAUAUGAAA-3′) [44], Mad2 SMARTpool (5′-GAAAGAUGGCAGUUUGAUA-3′, 5′-UAAAUAAUGUGGUGGAACA-3′, 5′-GAAAUCCGUUCAGUGAUCA-3′, 5′-UUACUCGAGUGCAGAAAUA-3′) [45] Wapl SMARTpool (5′-GGAGUAUAGUGCUCGGAAU-3′, 5′-GAGAGAUGUUUACGAGUUU-3′, 5′-CAAACAGUGAAUCGAGUAA-3′, 5′-CCAAAGAUACACGGGAUUA-3′), Tao1 si4 (5′-GUAAUAUGGUCCUUUCUAA-3′) (used as a negative control, [45]), Apc2 SMARTpool (5′-GAGAAGAAGUCCACACUAU-3′, 5′-GAUCGUAUCUACAACAUGC-3′, 5′-GACAUCAUCACCCUCUAUA-3′, 5′-GAGAUGAUCCAGCGUCUGU-3′) and p31 si1-3 (5′-GGUAUGAGAAGUCCGAAGA-3′, 5′-GGACACUAGUACCGCGAGU-3′, 5′-GAAGAUUGGUUUCGACCCA-3′) [9].

### Time-lapse Microscopy

Synchronised HeLa cells expressing GFP-histone H2B were released for 4 hours, treated with different drug combinations and imaged as described previously [18]. For analysing cyclin B degradation, cells were co-transfected with plasmids coding for cyclin B-Venus and DsRed-histone H2B using lipofectamine 2000 (Invitrogen). Then, cells were synchronised with a single thymidine block, released and imaged as described before [46].

### Antibody Techniques

Immunoblots were performed essentially as described [47], using the following antibodies: mouse anti-Apc2 (a gift from Jan-Michael Peters); sheep anti-Bub3 (A.J. Holland and S.S.T., unpublished); sheep anti-BubR1 [48]; E-7 mouse anti-Cdc20 (Santa Cruz Biotechnology); rabbit anti-Cdc27 [49]; mouse anti-cyclin A (E23, CRUK); rabbit anti-cyclin B1 (Sigma); sheep anti-Mad2 [44]; sheep anti-p31^comet^ [9]; rabbit anti-phospho histone H3 Ser10 (Milipore) and rabbit anti-Wapl (a gift from Jan-Michael Peters). Blots were visualised using a Biospecturm 500 imaging system (UVP) and quantified using a VisionWorks LS software (UVP) [49]. Immunofluoresence was done as described previously [50], using sheep anti-Bub1 [48] and rabbit anti-Smc3 (a gift from Jan-Michael Peters). Immunoprecipitations were performed as described [51].

## Supporting Information

Figure S1 **proTAME causes metaphase delay and cohesion fatigue. (A, B and C)** Scatter plot showing the amount of time cells take from NEBD to metaphase (A), metaphase to mitotic exit (B) and NEBD to cohesion fatigue (C) in the presence of proTAME. Note that this is the same data as in Fig. 1C. **(D)** Scatter plot showing the amount of time cells take from NEBD to mitotic exit in the presence of the proTAME compound described in this study (SST) or the one described Zeng et al (2010) (RK). Note that both compounds induced very similar phenotypes when used at the same concentration. P values were calculated using two-tailed Mann- Whitney test.
(TIF)

Figure S2 **Inhibition of SAC activity blocks the proTAME-induced mitotic arrest. (A)** Immunoblots of HeLa cells depleted of Mad2 or BubR1 by RNAi. Bub3 was used as a loading control. **(B)** Scatter plot showing the amount of time cells take from NEBD to metaphase in the presence of 20 µM proTAME and depletion of either BubR1 or Mad2. P values were calculated using two-tailed Mann-Whitney test.
(TIF)

Figure S3 **Proteasome inhibition increases the amount of MCC by stabilising Cdc20.** Immunoblots of immune complexes isolated from mitotic HeLa cells in the presence of absence of the proteasome inhibitor MG132. Note that, in the presence of MG132, Cdc20 is stabilised and thus, more Cdc20 immunocomplexes are obtained.
(TIF)
