# Supplementary figures and images for: Cohesion Fatigue Explains Why Pharmacological Inhibition of the APC/C Induces a Spindle Checkpoint-Dependent Mitotic Arrest
Source: PLoS One. 2012 Nov 7;7(11):e49041. doi: 10.1371/journal.pone.0049041 (PMC3492190; doi:10.1371/journal.pone.0049041)

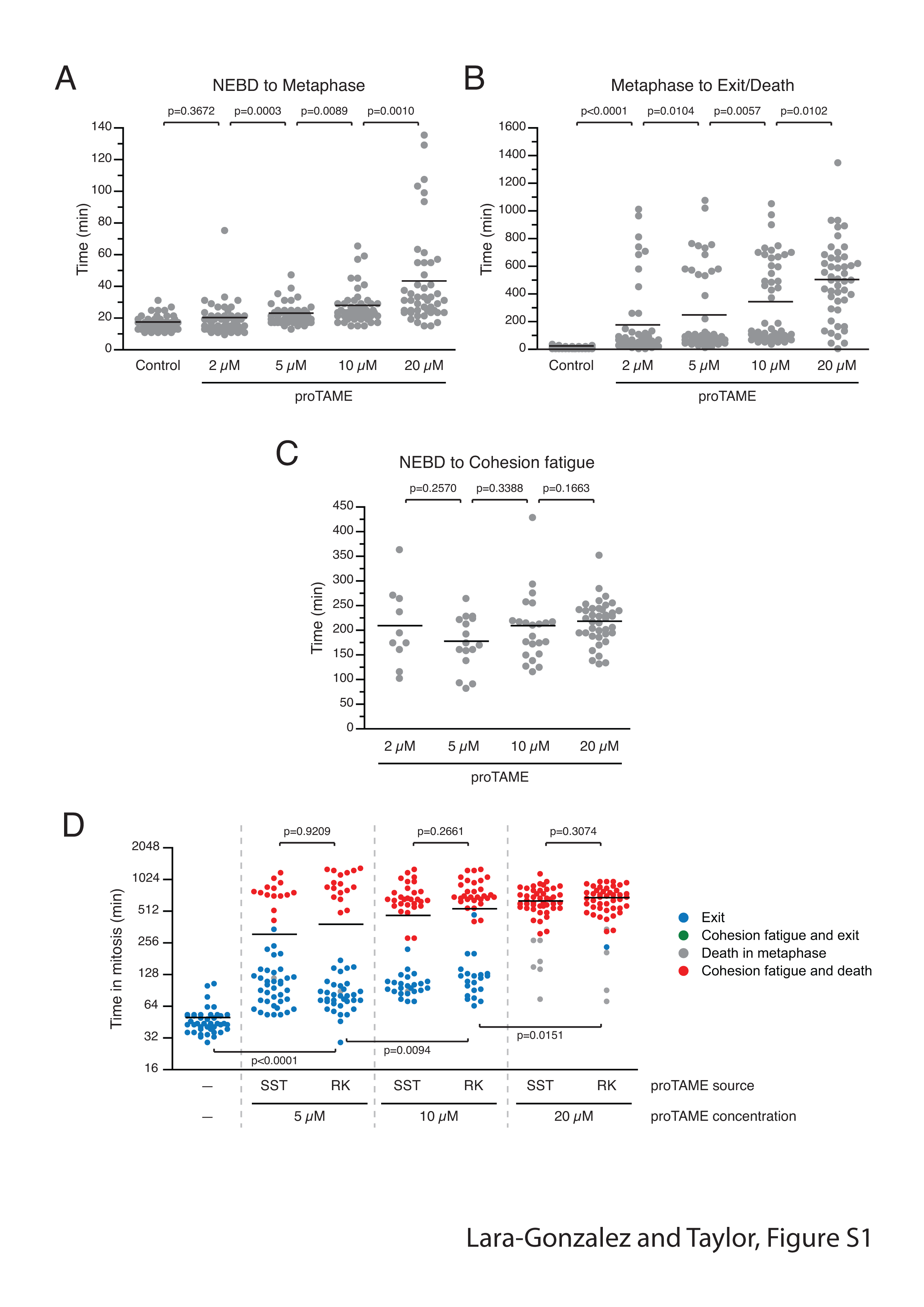

Supplement: Figure S1 — proTAME causes metaphase delay and cohesion fatigue. (A, B and C) Scatter plot showing the amount of time cells take from NEBD to metaphase (A), metaphase to mitotic exit (B) and NEBD to cohesion fatigue (C) in the presence of proTAME. Note that this is the same data as in Fig. 1C. (D) Scatter plot showing the amount of time cells take from NEBD to mitotic exit in the presence of the proTAME compound described in this study (SST) or the one described Zeng et al (2010) (RK). Note that both compounds induced very similar phenotypes when used at the same concentration. P values were calculated using two-tailed Mann- Whitney test. (TIF) [file pone.0049041.s001.tif]

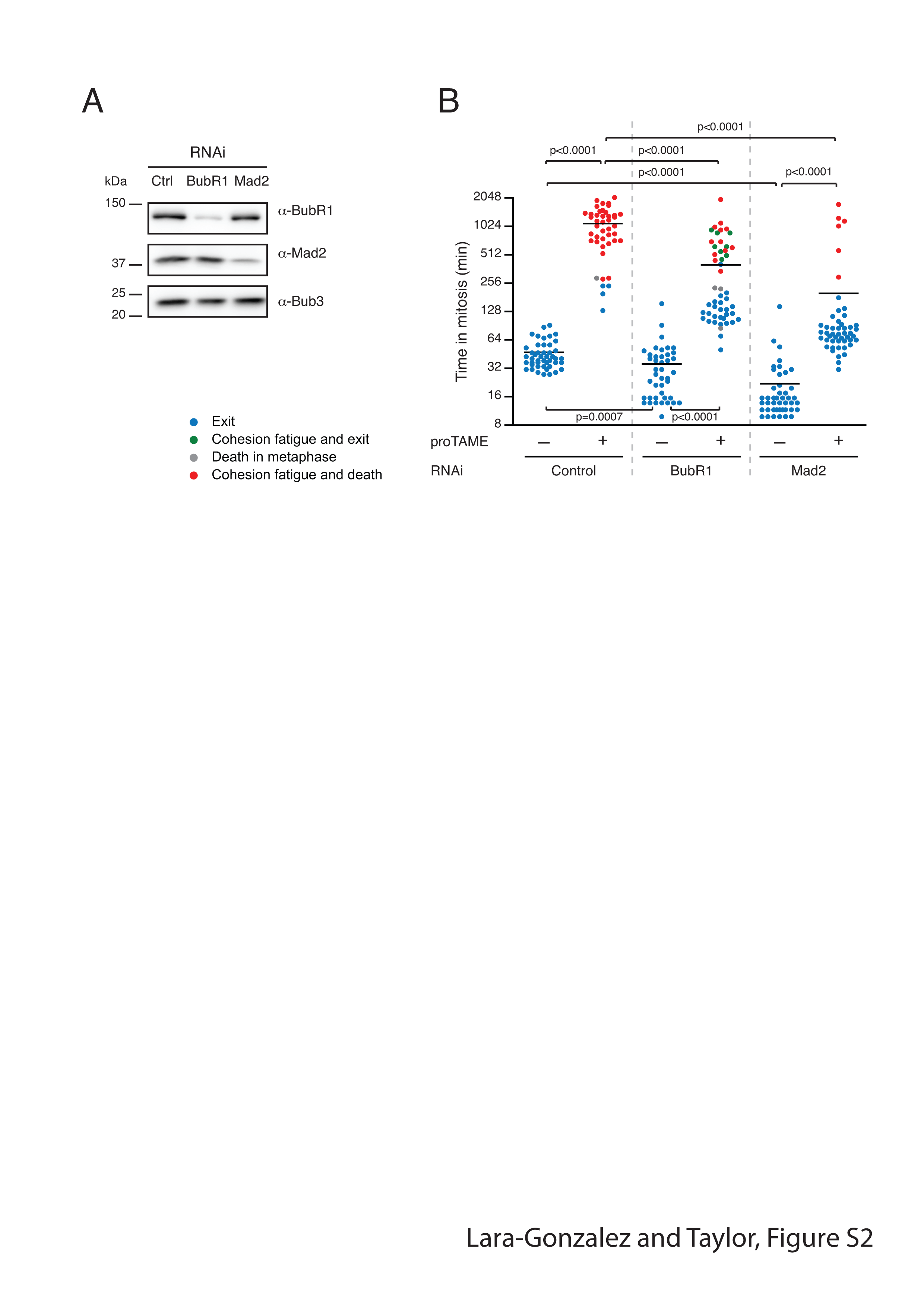

Supplement: Figure S2 — Inhibition of SAC activity blocks the proTAME-induced mitotic arrest. (A) Immunoblots of HeLa cells depleted of Mad2 or BubR1 by RNAi. Bub3 was used as a loading control. (B) Scatter plot showing the amount of time cells take from NEBD to metaphase in the presence of 20 µM proTAME and depletion of either BubR1 or Mad2. P values were calculated using two-tailed Mann-Whitney test. (TIF) [file pone.0049041.s002.tif]

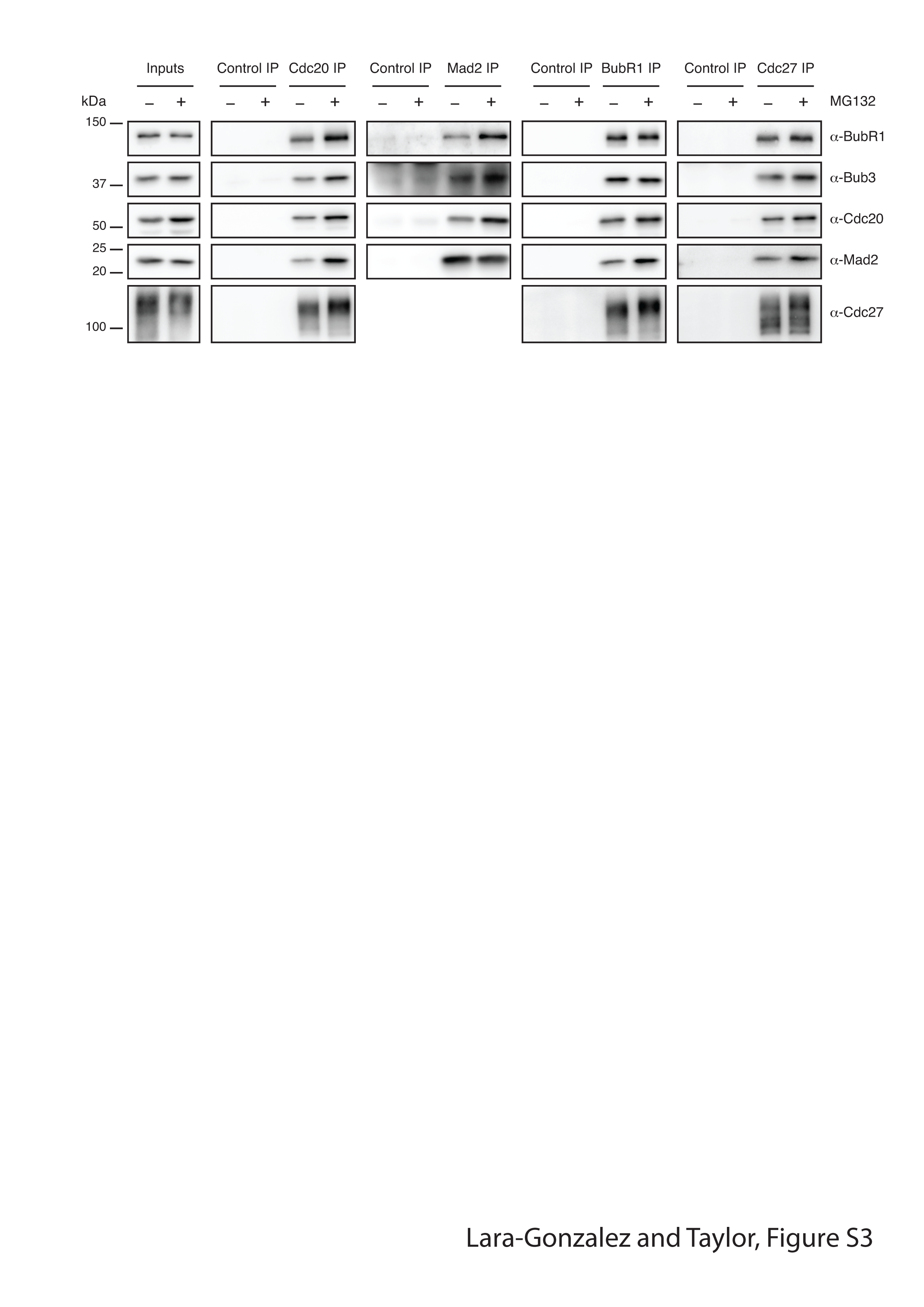

Supplement: Figure S3 — Proteasome inhibition increases the amount of MCC by stabilising Cdc20. Immunoblots of immune complexes isolated from mitotic HeLa cells in the presence of absence of the proteasome inhibitor MG132. Note that, in the presence of MG132, Cdc20 is stabilised and thus, more Cdc20 immunocomplexes are obtained. (TIF) [file pone.0049041.s003.tif]
